# Effect of *BCHE* single nucleotide polymorphisms on lipid metabolism markers in women

**DOI:** 10.1590/1678-4685-GMB-2016-0123

**Published:** 2017-05-11

**Authors:** Jéssica de Oliveira, Luciane Viater Tureck, Willian dos Santos, Louise Farah Saliba, Caroline Schovanz Schenknecht, Débora Scaraboto, Ricardo Lehtonen R. Souza, Lupe Furtado-Alle

**Affiliations:** 1 Departamento de Genética, Universidade Federal do Paraná (UFPR), Curitiba, PR, Brazil; 2 Departamento de Nutrição, Pontifícia Universidade Católica do Paraná (PUC-PR), Curitiba, PR, Brazil; 3 Departamento Acadêmico de Ensino, Universidade Tecnológica Federal do Paraná (UTFPR), Ponta Grossa, PR, Brazil

**Keywords:** *BCHE* gene, obesity, lipid metabolism, polymorphisms

## Abstract

Butyrylcholinesterase (BChE) activity and polymorphisms in its encoding gene had previously been associated with metabolic traits of obesity. This study investigated the association of three single nucleotide polymorphisms (SNPs) in the *BCHE* gene: -116G > A (rs1126680), 1615GA (rs1803274), 1914A < G (rs3495), with obesity and lipid metabolism markers, body mass index (BMI), total cholesterol (TC), low density lipoprotein cholesterol (LDL-C), high density lipoprotein cholesterol (HDL-C), triglyceride (TG) levels, and BChE enzymatic activity in obese (BMI≥30/n = 226) and non-obese women (BMI < 25/n = 81). *BCHE* SNPs genotyping was obtained by TaqMan allelic discrimination assay and by RFLP-PCR. Plasmatic BChE activity was measured using propionylthiocholine as substrate. Similar allele frequencies were found in obese and non-obese women for the three studied SNPs (p > 0.05). The dominant and recessive models were tested, and different effects were found. The -116A allele showed a dominant effect in BChE activity reduction in both non-obese and obese women (p = 0.045 and p < 0.001, respectively). The 1914A > G and 1615GA SNPs influenced the TG levels only in obese women. The 1914G and the 1615A alleles were associated with decreased plasma levels of TG. Thus, our results suggest that the obesity condition, characterized by loss of energy homeostasis, is modulated by BCHE polymorphisms.

## Introduction

The human butyrylcholinesterase (BChE, EC 3.1.1.8), encoded by the *BCHE* gene (3q26.1-q26.2), is a cholinesterase synthesized in the liver and found in plasma, pancreas, liver, skin, smooth muscle, endothelium, brain and heart (Wescoe *et al*., 1947; Chatonnet and Lockridge, 1989). Although it is able of hydrolyzing acetylcholine similar to AChE, BChE functions appear to be more varied and remain not fully understood (Valle *et al*., 2011). BChE activity is heritable (H^2^ = 81.4 ± 2.8%, p = 1.0910^−32^), influenced by *BCHE* gene polymorphisms (Valle *et al*., 2011), and associated with lipid metabolism and factors related to obesity, such as weight (Chautard-Freire-Maia *et al*., 1991), body mass index (BMI) (Alcântara *et al*., 2005; Valle *et al*., 2011; Silva *et al*., 2012; Lima *et al*., 2013; Milano *et al*., 2013) and lipid profile (Alcântara *et al*., 2002; Benyamin *et al*., 2011, Scacchi *et al*., 2011; Chaves *et al*., 2013; Lima *et al*., 2013).

The association of *BCHE* gene polymorphisms with obesity and related parameters has been demonstrated by many studies. *BCHE* knockout mice become obese when treated with a high-fat diet similar to that given to wild-type mice (Li *et al*., 2008). Furthermore, people with high BChE activity have lower BMI (Chautard-Freire-Maia *et al*., 1991). Thus, the influence of *BCHE* polymorphisms influence may be direct, through enzymatic activity variation, or indirect, through changes in the interactions between BChE and other proteins.

Three *BCHE* SNPs seem to have important functional effects in this context: -116G > A (rs1803274), 1615GA (rs1126680, *K variant; p. A539T*), and 1914A > G (rs3495), that are in linkage disequilibrium, preferentially found in *cis* configuration (Bartels *et al*., 1990) (D′ = 1 for the three loci; and *R* ^2^ = 0.547 (-116G > A and 1914A > G); *R* ^2^ = 0.208 (1615GA and 1914A > G); *R* ^2^ = 0.380 (-116G > A and 1615GA); data from Haploview 4.1 software) (Barrett *et al*., 2005). According to Furtado-Alle *et al*. (2008), the concomitant presence of -116A and 1615A variants was responsible for most of the variance in BMI and BChE activity reduction. The 1914G variant was also associated with BChE activity decrease and higher mean BMI and triglyceride levels (Lima *et al*., 2013).

Here we evaluated the effects of these three *BCHE* gene SNPs on enzyme activity, lipid metabolism and BMI. To examine these possible effects, we tested dominant (-116G > A) and recessive (1615GA and 1914A > G) genetic models on BChE activity and lipid metabolism markers in obese and non-obese women from Southern Brazil.

## Material and Methods

### Samples

The sample consisted of 307 adult women, self-declared Euro-Brazilian, 226 of which were classified as obese (BMI≥30 kg/m^2^) and 81 as non-obese (BMI < 25 kg/m^2^). Weight and height were measured with an accuracy of 0.1 kg and 0.1 cm, respectively.

Women interested in participating voluntarily in the study were evaluated by a professional team of nutritionists, nurses and geneticists. Criteria for inclusion were: age ≥ 20 years, apparent health, not pregnant, not breastfeeding, and before menopause. The study excluded women who were on diet and under treatment with weight loss medication, vegetarian, suffering from type 1 diabetes, with untreated hypothyroidism, renal chronic disease and other chronic diseases.

Twelve-hour fast blood was collected from participants, and triglycerides (TG), total cholesterol (TC), high density lipoprotein cholesterol (HDL-C) and low density lipoprotein cholesterol (LDL-C) were measured by standard automated methods.

The study was approved by the ethics committee of the Federal University of Paraná (CEP/SD 1159.084.11.06/CAAE0082.0.091.000-11), and by the ethics committee of the Pontifical Catholic University of Paraná (0005306/11).

### DNA and plasma BChE analysis

DNA was extracted from peripheral blood by a modified salting-out method (Lahiri and Nurnberger Jr, 1991), and diluted to the final concentration of 20 ng/μL and 100 ng/μL for TaqMan and restriction fragment length polymorphism polymerase chain reaction (RFLP-PCR) genotyping, respectively. Genotyping of -116G > A and 1615GA sites were obtained by a TaqMan allelic discrimination assay (Applied Biosystems), according to the following steps: (1) 50 °C for 2 min., (2) 95 °C for 10 min., (3) 50 cycles of 95 °C for 15 s and 62 °C for 1 min., (4) 60 °C for 30 s.

The RFLP-PCR for the 1914A > G site genotyping used the following pair of primers: 5′AGCAAGAAAGA AAGTTGTGTGGGTCT3′ and 3′AGCAGAGCACTGT AATTTTGGGGG5′, generating a fragment of 298 bp. Amplification cycles were: (1) 95 °C for 30 s, (2) 95 °C for 30 s, (3) 55 °C for 30 s, (4) 72 °C for 30 s, (5) 35 cycles repeating the steps 2 to 4, (6) 72 °C for 10 min. After amplification, the DNA was incubated for 15 h at 37 °C with *Xce*l (*Nsp*l) restriction enzyme (Thermo Scientific), as recommended by the manufacturer. The enzyme cleaves the site in the presence of the 1914A allele, generating two fragments (195 bp and 103 bp). The fragments were analyzed by electrophoresis at 4 °C (250 V, 35 mA and 60 W for 90 min) in polyacrylamide gels (8%), followed by gel staining with silver nitrate (Budowle *et al*., 1991).

Plasmatic BChE activity was measured using propionylthiocholine as substrate at −25 °C (Dietz *et al*., 1972).

### Statistical analysis

Allele and genotype frequencies were obtained by direct counting, and allele frequency comparisons between groups were performed by the χ2 test, as well as Hardy-Weinberg equilibrium verification. The normal distribution of variables was tested by the Kolmogorov-Smirnov test with Lilliefors correction. Comparisons between means were made by Student's *t*-test for unpaired variables with parametric distribution (TC) and Mann-Whitney test for variables with non-parametric distribution (BMI, BChE activity, and HDL-C, LDL-C and TG levels). Multiple regression analysis was performed for parametric variables, and Spearman's rank correlation analysis for non-parametric variables. The probability value for the comparative tests were considered significant at p < 0.05 (5%).

## Results

Genotype and allele frequencies in obese and non-obese women and between-group comparisons are shown in Table 1. The alleles were shown to be equally distributed between obese and non-obese women (p > 0.05), and all genotype distributions were in Hardy-Weinberg equilibrium (p > 0.05).

**Table 1:** Comparisons of allele frequencies (p) and genotype distribution of -116G > A, 1615AG and 1914A > G SNPs (mean % ± standard error) in obese and non-obese women.

SNP -116G > A						
Groups	Allele frequencies			Genotype frequencies		
	A	G	p	GG	GA	AA
Obese	8 ± 1.2%	92 ± 1.2%	0.777	85.4% (n = 193)	13.3% (n = 30)	1.3% (n = 3)
Non-obese	8 ± 2.0%	92 ± 2.0%	84% (n = 68)	16% (n = 13)	0% (n = 0)	
**SNP 1615GA**						
**Groups**	**Allele frequencies**			**Genotype frequencies**		
	**A**	**G**	**p**	**GG**	**GA**	**AA**
Obese	19 ± 1.8%	81 ± 1.8%	0.591	65% (n = 147)	33% (n = 74)	2% (n = 5)
Non-obese	22 ± 3.0%	78 ± 3.0%	64.2% (n = 52)	28.4% (n = 23)	7.4% (n = 6)	
**SNP 1914A > G**						
**Groups**	**Allele frequencies**			**Genotype frequencies**		
	**A**	**G**	**p**	**AA**	**AG**	**GG**
Obese	65 ± 2.2%	35 ± 2.2%	0.833	42.9% (n = 97)	43.4% (n = 98)	13.7% (n = 31)
Non-obese	63 ± 4.0%	37 ± 4.0%	45.7% (n = 37)	34.6% (n = 28)	19.7% (n = 16)	

Regardless of genotype, obese women showed similar BChE activity and lipid metabolism markers to non-obese women (p > 0.05), except for the triglycerides mean level, which was higher among obese women (p = 0.001) (Table 2).

**Table 2:** Comparisons of lipid metabolism markers (mean ± standard error) among obese and non-obese women.

Variable	Mean obese (n = 226)	Mean non-obese (n = 81)	p
BChE activity (kU/L)	5.19 ± 0.11	5.17 ± 0.20	0.586
HDL-C (mg/dL)	51.38 ± 0.87	53.17 ± 1.69	0.421
LDL-C (mg/dL)	115.24 ± 1.94	117.10 ± 4.04	0.320
TG (mg/dL)	142.22 ± 4.54	105.36 ± 6.50	**0.001** *
TC (mg/dL)	195.10 ± 2.30	188.38 ± 5.80	0.202

* Significant value in bold type. BChE: Butyrylcholinesterase TG: triglycerides, TC: total cholesterol, HDL-C: high density lipoprotein cholesterol, LDL-C: low density lipoprotein cholesterol.

For the following analysis, the -116G > A genotypes were grouped according to the dominant model (GA + AA), due to low frequency of the A allele. The 1615GA and 1914A > G genotypes were grouped according to the recessive model (GG+GA and AA+AG respectively), due to the effect of each genotype when analyzed separately. The effects of these models on BChE activity and lipid profile markers were tested in non-obese (Table 3) and obese women (Table 4).

**Table 3:** Anthropometric and lipid metabolism markers (mean ± standard error) and comparisons in non-obese women stratified by dominant and recessive models of *BCHE* gene SNPs.

NON OBESE									
	SNP-116G > A		p	SNP1615GA		p	SNP 1914 A > G		p
	GG	GA + AA		GG + GA	AA		AA + AG	GG	
	(n = 68)	(n = 13)		(n = 75)	(n = 6)		(n = 65)	(n = 16)	
BMI (kg/m^2^)	22.27 ± 0.23	22.04 ± 0.54	0.652	22.23 ± 0.27	22.51 ± 0.74		22.20 ± 0.33	22.19 ± 0.42	
						0.830			0.948
BChE activity (kU/L)	5.40 ± 0.24	4.19 ± 0.44	0.045	5.28 ± 0.28	4.22 ± 0.87		5.40 ± 0.32	4.78 ± 0.36	
						0.249			0.660
HDL-C (mg/dL)	53.77 ± 1.86	50.4 ± 4.34	0.337	53.11 ± 2.07	53.80 ± 7.44		52.542.58	57 ± 4.55	
						1.00			0.456
LDL-C (mg/dL)	118.62 ± 4.62	106.3 ± 5.71	0.144	117.29 ± 4.96	115.76 ± 8.68		118.11 ± 5.66	108.06 ± 10.77	
						0.855			0.598
TG (mg/dL)	101.46 ± 6.36	120.9 ± 25.43	0.804	101.59 ± 6.89	157.40 ± 36.98		101.24 ± 8.34	106.23 ± 18.78	
						0.165			0.911
TC (mg/dL)	189.35 ± 6.65	179.9 ± 9.35	0.584	187.46 ± 7.00	201 ± 6.70		188.05 ± 7.53	181.92 ± 17.20	
						0.562			0.694

Significant values in bold type. BChE: Butyrylcholinesterase; BMI: body mass index; TG: triglycerides; TC: total cholesterol; HDL-C: high density lipoprotein cholesterol; LDL-C: low density lipoprotein cholesterol.

**Table 4:** Anthropometric and lipid metabolism markers (mean ± standard error) and comparisons in obese women stratified by dominant and recessive models of *BCHE* gene SNPs.

OBESE									
Variables	-116 G > A		p	1615GA		p	1914A > G		p
	GG (n = 193)	GA + AA (n = 33)		GG + GA (n = 221)	AA (n = 5)		AA + AG (n = 195)	GG (n = 31)	
BMI (kg/m^2^)	35.39 ± 0.38	34.32 ± 0.67	0.522	35.28 ± 0.42	34.56 ± 2.09		35.52 ± 0.57	34.07 ± 0.65	
						0.544			0.202
BChE activity (kU/L)	5.37 ± 0.12	4.32 ± 0.25	0.00003	5.21 ± 0.15	5.65+1.15		5.07 ± 0.17	5.80+0.14	
						0.789			0.018
HDL-C (mg/dL)	50.55 ± 0.89	52.3 ± 2.32	0.580	50.98 ± 1.11	51.43+5.65		51.23 ± 1.39	49.53+2.00	
						0.780			0.560
LDL-C (mg/dL)	114.39 ± 2.18	119.67 ± 4.93	0.310	115.04 ± 2.63	124.73+8.10		114.11 ± 3.20	121.19+5.86	
						0.267			0.222
TG (mg/dL)	144.26 ± 5.31	124.57 ± 8.31	0.118	142.92 ± 6.54	95.57+8.52		145.12 ± 6.55	117.18+10.5	
						0.019			0.015
TC (mg/dL)	193.85 ± 2.57	196.86 ± 5.85	0.639	194.64 ± 3.07	195.28+10.83		194.41 ± 3.60	194.21+6.65	
						0.963			0.978

Significant values in bold type. BChE: Butyrylcholinesterase; BMI: body mass index; TG: triglycerides; TC: total cholesterol; HDL-C: high density lipoprotein cholesterol; LDL-C: low density lipoprotein cholesterol.

In non-obese women only the -116G > A SNP showed a significant effect: the -116A carriers (dominant model) had significantly lower mean BChE activity, compared with non-carriers (GG) (p = 0.045) (Table 3). Among obese women, in addition to the -116A dominant effect of lowering BChE activity (p < 0.001), the recessive effects of 1615GA and 1914A > G SNPs in the reduction of triglyceride levels were identified. The less common homozygous genotype of 1615GA and 1914A > G SNPs (AA and GG, respectively) showed lower triglyceride mean levels compared with the grouped heterozygous and common homozygous genotypes (p = 0.019 and p = 0.015, respectively) (Table 4). The obese carriers of 1914A > G homozygous genotype (GG) also showed higher BChE activity compared with carriers of other genotypes (Table 4).

Multiple regression analysis confirmed the independent effect of -116G > A SNP on mean BChE activity among non-obese women (p = 0.048) (Table 5). Among obese women, the independent effect of -116G > A SNP and BMI on the mean BChE activity were confirmed (p = 0.010 and p = 0.027 respectively) (Table 5). Multiple regression analysis also confirmed that, among obese women, the 1914A > G polymorphism acted independently on triglyceride levels (p = 0.024) (Table 5). A significant and positive correlation between BChE activity and TG levels in obese women (ρ = 0.1726, p = 0.0076) was found by Spearman's correlation analysis.

**Table 5:** Multiple regression analysis results

Group	Dependent variable	Independent variables considered	Independent variable confirmed	β ± standard error	p
Non-obese	BChE activity	-116G > A and BMI	-116G > A	−0.218 ± 0.108	0.048
Obese	BChE activity	-116G > A and BMI	-116G > A and BMI	(−0.189 ± 0.073), (−0.167 ± 0.073), respectively	0.010 and 0.023, respectively
Obese	TG	1914A > G and BChE activity	1914G > A and BChE activity	(−0.155 ± 0.068), (0.210 ± 0.068), respectively	0.024 and 0.002, respectively

Significant values in bold type. BChE: Butyrylcholinesterase

Considering the linkage disequilibrium between the three sites, a combined genotype analysis was conducted. Significant differences in BChE activity and TG levels remained in obese women only. The less frequent allele combinations, considering all three sites (Table S1), and two combined sites: 1615G A and 1914A > G (Table S2), -116G > A and 1914A > G (Table S3) and -116G > A and 1615GA (Table S4) showed lower means of BChE activity and TG levels (p < 0.05).

## Discussion

The results presented above suggest that the 1615GA and 1914A > G polymorphisms are associated with changes in triglyceride levels in obese women. However, only the 1914A > G independent effect was confirmed by regression analysis, which may indicate that differences in TG mean levels between 1615GA genotypes were due to the linkage disequilibrium between these two sites.

The 1914A > G influence on TG levels has been described in the literature (Lima *et al*., 2013), besides the strong positive correlation between BChE activity and TG levels (Iwasaki *et al*., 2004; Benyamin *et al*., 2011), which was also found in our study.

The relationship between BChE and TG levels may be caused by the fatty acid increase from adipose tissue due to BChE hepatic synthesis, which is consistent with higher TG levels, ultimately leading to higher BChE activity (Cucuiani *et al*., 2002). In addition, it was suggested that hyperlipidemia leads to changes in the tertiary and quaternary BChE structure (Kálmán *et al*., 2004). Other factors must be considered in this context, such as the possible effect of other genes that are in the interface between lipid and carbohydrate metabolism that may increase the metabolic risk profile and, thus, indirectly affect BChE activity through TG levels (Benyamin *et al*., 2011).

The molecular mechanism of this association remains uncertain, as well as whether BChE activity variation is caused by metabolic abnormalities, or if this metabolic disorder is secondary to altered BChE activity. It is probably a feedback system, therefore it is both the cause and the effect. This was suggested by Silva *et al*. (2012), whose study showed the physiological regularization of BChE activity after a physical exercise program, where the BChE activity and lipid profile became normal in response to exercise.

The polymorphisms' effects on BChE activity and TG levels in obese women seem to be independent, since the -116G > A polymorphism acted on BChE activity according to the dominant model, while the 1914A > G acted in a recessive form on TG levels. Differently from Lima *et al*., 2013, we found no 1914G allele association with obesity, as there was no difference in allele frequencies between obese and non-obese women, in our study the 1914A > G polymorphism effect on TG levels differed between groups. This discrepancy may be due to differences between the samples. The study of Lima *et al*. (2013) was based on a population sample, therefore it was heterogeneous, composed of obese and non-obese men and women. In our study we restricted our analysis to obese women. Specific metabolic conditions associated with obesity and the influence of sex hormones, especially estrogen, on the lipid profile (Bataille *et al*., 2005), can modulate the genetic polymorphisms' effect differently, as observed in previous studies (Ordovas, 2008; Tureck *et al*., 2014; Locke,2015).

In the evaluation of BChE activity, obesity is a major condition to be considered, since several studies showed that obese individuals tend to have increased activity of this enzyme as a result of increased levels of choline esters, which are products of free fatty acid metabolism and hepatic lipogenesis, and both metabolic traits are altered with obesity (Alcântara *et al*., 2005; Randell *et al*., 2005; Furtado-Alle *et al*., 2008). In our study, however, there was no significant difference in mean BChE activity between obese and non-obese women. This may be due to lipid profile similarities among these women, since only the mean TG level was higher among obese compared to non-obese. This suggests that the excess fat tissue itself is not a determinant factor for the increase in BChE activity, and that a metabolic disorder with an unfavorable lipid profile is more important in this regard. Iwasaki *et al*. (2004) evaluated the degree of hepatic steatosis based on BMI and liver function markers of liver donors, and found that obese patients without liver steatosis had normal BChE activity levels, whereas both obese as well as non-obese with this condition showed an increased BChE activity, which strengthens our hypothesis.

Besides the influence of these endogenous factors, polymorphisms in the *BCHE* gene are also associated with BChE activity variation (Benyamin *et al*., 2011). Our findings suggest that the -116A allele was responsible for lower levels of enzymatic activity in both obese and non-obese women. This result was similar to that found by Furtado-Alle *et al*. (2008), who found a decreased BChE activity in -116A allele carriers in obese and non-obese men. The -116G > A independent effect on BChE activity was confirmed in our study by multiple regression analysis in both groups. However, BMI was an independent factor for this variable only in the obese group. The relative BMI contribution to the BChE activity appears to respond to internal metabolic factors and in homeostasis imbalance situations, such as caused by obesity.

Certain limitation should be highlighted for this study, such as the small number of samples, especially in the control group, and the exclusion of men, which could have revealed a possible gender influence.

In conclusion, an unfavorable lipid status seems to be a determining factor in BChE enzymatic activity. In addition, the -116G > A and 1914A > G polymorphisms influence both BChE activity and TG levels, the -116G > A dominant effect on the BChE activity is independent of obesity status, and the 1914A > G recessive effect on the TG levels is obesity-dependent.

## Supplementary Material

The following online material is available for this article:

Table S1 - Anthropometric and biochemical variables (mean ± standard error) in obese and non-obese women stratified by usual homozygous and less frequent alleles carriers for -116G > A, 1615GA and 1914A > G SNPs.

Table S2 - Anthropometric and biochemical variables (mean ± standard error) in obese and non-obese women stratified by usual homozygous and less frequent alleles carriers for 1615GA and 1914A > G SNPs.

Table S3 - Anthropometric and biochemical variables (mean ± standard error) in obese and non-obese women stratified by usual homozygous and less frequent alleles carriers for -116G > A and 1914A > G SNPs.

Table S4 - Anthropometric and biochemical variables (mean ± standard error) in obese and non-obese women stratified by usual homozygous and less frequent alleles carriers for -116G > A and 1615GA SNPs.
