# Supplementary material for: Effect of BCHE single nucleotide polymorphisms on lipid metabolism markers in women
Source: Genet Mol Biol. 2017 May 11;40(2):408–14. doi: 10.1590/1678-4685-GMB-2016-0123 (PMC5488457; doi:10.1590/1678-4685-GMB-2016-0123)
Supplement: Supplementary file 1 [file 1415-4757-gmb-1678-4685-GMB-2016-0123-Suppl01.pdf]

Table S1 – Anthropometric and biochemical variables (mean ± standard error) in obese and non-obese women stratified by usual homozygous and less frequent alleles carriers for -116G>A, 1615G>A and 1914A>G SNPs.

	**Obese**			**Non-obese**		
**Parameter**	116AA + 1615AA + 1914GG	(-116AG+AA) + (1615AG+AA) + (1914AG+GG)	p	116AA + 1615AA + 1914GG	(-116AG+AA) + (1615AG+AA) + (1914AG+GG)	p
	(n = 81)	(n = 21)		(n = 32)	(n = 10)	
BMI (kg/m²)	35.53 ± 0.61	34.37 ± 0.73	0.695	21.94 ± 0.35	22.04 ± 0.54	0.916
BChE activity (kU/L)	5.37 ± 0.18	4.59 ± 0.35	**0.009**	5.23 ± 0.33	4.19 ± 0.44	0.175
HDL-C (mg/dL)	52.55 ± 1.58	51.24 ± 2.94	0.670	54.63 ± 2.70	50.4± 4.34	0.440
LDL-C (mg/dL)	113.00± 3.42	123.25 ± 6.64	0.164	120.01 ± 5.84	106.3 ± 5.71	0.219
TG (mg/dL)	148.22 ± 6.87	121.95 ± 11.40	**0.026**	93.41 ± 6.63	120.9 ± 25.43	0.937
TC (mg/dL)	194.99 ± 3.90	198.85 ± 7.99	0.658	190.06 ± 7.86	179.9 ± 9.35	0.322

BChE: Butyrylcholinesterase; BMI: body mass index; TG: triglycerides; TC: total cholesterol; HDL-C: high density lipoprotein cholesterol; LDL-C: low density lipoprotein cholesterol.
