# Supplementary material for: Effect of BCHE single nucleotide polymorphisms on lipid metabolism markers in women
Source: Genet Mol Biol. 2017 May 11;40(2):408–14. doi: 10.1590/1678-4685-GMB-2016-0123 (PMC5488457; doi:10.1590/1678-4685-GMB-2016-0123)
Supplement: Supplementary file 2 [file 1415-4757-gmb-1678-4685-GMB-2016-0123-Suppl02.pdf]

Table S2 - Anthropometric and biochemical variables (mean ± standard error) in obese and non-obese women stratified by usual homozygous and less frequent alleles carriers for 1615G>A and 1914A>G SNPs.

Parameter	Obese			Non-obese		
	1615AA + 1914GG (n = 86)	(1615AG+AA) + (1914AG+GG) (n = 64)	p	1615AA + 1914GG (n = 32)	(1615AG+AA) + (1914AG+GG) (n = 10)	p
BMI (kg/m²)	35.51 ± 0.6	35.96 ± 0.64	0.469	21.93 ± 0.35	22.37 ± 0.35	0.378
BChE activity (kU/L)	5.34 ± 0.17	4.91 ± 0.20	**0.039**	5.23 ± 0.33	4.98 ± 0.35	0.850
HDL-C (mg/dL)	52.63 ± 1.49	49.08 ± 1.39	0.202	54.63 ± 2.70	54.6 ± 3.31	0.816
LDL-C (mg/dL)	113.12 ± 3.34	117.7 ± 3.62	0.322	120.01 ± 5.84	107.21 ± 7.34	0.422
TG (mg/dL)	146.95 ± 6.57	124.63 ± 6.22	**0.014**	93.41 ± 6.63	107.47 ± 12.93	0.401
TC (mg/dL)	194.94 ± 3.8	191.6 ± 4.33	0.563	190.06 ± 7.86	180.24 ± 11.67	0.471

BChE: Butyrylcholinesterase; BMI: body mass index; TG: triglycerides; TC: total cholesterol; HDL-C: high density lipoprotein cholesterol; LDL-C: low density lipoprotein cholesterol.
