# Supplementary material for: Effect of BCHE single nucleotide polymorphisms on lipid metabolism markers in women
Source: Genet Mol Biol. 2017 May 11;40(2):408–14. doi: 10.1590/1678-4685-GMB-2016-0123 (PMC5488457; doi:10.1590/1678-4685-GMB-2016-0123)
Supplement: Supplementary file 3 [file 1415-4757-gmb-1678-4685-GMB-2016-0123-Suppl03.pdf]

Table S3 - Anthropometric and biochemical variables (mean ± standard error) in obese and non-obese women stratified by usual homozygous and less frequent alleles carriers for -116G>A and 1914A>G SNPs.

	**Obese**			**Non-obese**		
**Parameter**	-116AA + 1914GG (n = 88)	(-116AG+AA) + (1914AG+GG) (n = 25)	p	-116AA + 1914GG (n = 33)	(-116AG+AA) + (1914AG+GG) (n = 11)	p
BMI (kg/m²)	35.42 ± 0.59	35.96 ± 0.64	0.751	21.97 ± 0.39	21.84 ± 0.57	0.839
BChE activity (kU/L)	5.33 ± 0.17	4.91 ± 0.20	**0.002**	5.19 ± 0.32	4.30 ± 0.50	0.203
HDL-C (mg/dL)	52.57 ± 1.44	49.08 ± 1.39	0.948	54.27 ± 2.63	51.22 ± 4.76	0.487
LDL-C (mg/dL)	112.63 ± 3.29	117.7 ± 3.62	0.211	121.37 ± 5.82	103.67 ± 5.66	0.181
TG (mg/dL)	150.76 ± 6.76	124.63 ± 6.22	**0.046**	92.57 ± 6.48	103.33 ± 20.56	0.880
TC (mg/dL)	195.16 ± 3.66	191.6 ± 4.33	0.661	191. ± 7.67	174.44 ± 8.47	0.290

BChE: Butyrylcholinesterase; BMI: body mass index; TG: triglycerides; TC: total cholesterol; HDL-C: high density lipoprotein cholesterol; LDL-C: low density lipoprotein cholesterol.
