# Supplementary material for: Effect of BCHE single nucleotide polymorphisms on lipid metabolism markers in women
Source: Genet Mol Biol. 2017 May 11;40(2):408–14. doi: 10.1590/1678-4685-GMB-2016-0123 (PMC5488457; doi:10.1590/1678-4685-GMB-2016-0123)
Supplement: Supplementary file 4 [file 1415-4757-gmb-1678-4685-GMB-2016-0123-Suppl04.pdf]

Table S4 - Anthropometric and biochemical variables (mean ± standard error) in obese and non-obese women stratified by usual homozygous and less frequent alleles carriers for -116G>A and 1615G>A SNPs.

	Obese			Non-obese		
**Parameter**	-116AA + 1615AA (n = 139)	(-116AG+AA) + (1615AG+AA) (n = 28)	p	-116AA + 1615AA (n = 50)	(-116AG+AA) + (1615AG+AA) (n = 13)	p
BMI (kg/m²)	35.05 ± 0.43	34.21 ± 0.68	0.751	22.11 ± 0.26	22.04 ± 0.54	0.890
BChE activity (kU/L)	5.52 ± 0.15	4.39 ± 0.30	**0.000**	5.40 ± 0.28	4.2 ± 0.45	0.056
HDL-C (mg/dL)	51.6 ± 1.14	52.11 ± 2.77	0.907	53.18 ± 2.07	50.4 ± 4.34	0.419
LDL-C (mg/dL)	114.53 ± 2.68	122.34 ± 5.39	0.195	119.8 ± 4.96	106.3 ± 5.71	0.153
TG (mg/dL)	150.35 ± 6.81	122 ± 9.65	**0.043**	99.98 ± 6.89	120.9 ± 25.43	0.792
TC (mg/dL)	196.32 ± 3.15	198.82 ± 6.68	0.743	189.86 ± 7	179.9 ± 9.34	0.543
